# Noval advance of histone modification in inflammatory skin diseases and related treatment methods

**DOI:** 10.3389/fimmu.2023.1286776

**Published:** 2024-01-03

**Authors:** Lichen Zhang, Rongrong Chai, Zongguang Tai, Fengze Miao, Xinwei Shi, Zhongjian Chen, Quangang Zhu

**Affiliations:** ^1^ Shanghai Skin Disease Hospital, School of Medicine, Tongji University, Shanghai, China; ^2^ Shanghai Engineering Research Center of External Chinese Medicine, Shanghai, China

**Keywords:** histone modification, inflammatory skin disease, therapy, epigenetic, inflammatory reaction

## Abstract

Inflammatory skin diseases are a group of diseases caused by the disruption of skin tissue due to immune system disorders. Histone modification plays a pivotal role in the pathogenesis and treatment of chronic inflammatory skin diseases, encompassing a wide range of conditions, including psoriasis, atopic dermatitis, lupus, systemic sclerosis, contact dermatitis, lichen planus, and alopecia areata. Analyzing histone modification as a significant epigenetic regulatory approach holds great promise for advancing our understanding and managing these complex disorders. Additionally, therapeutic interventions targeting histone modifications have emerged as promising strategies for effectively managing inflammatory skin disorders. This comprehensive review provides an overview of the diverse types of histone modification. We discuss the intricate association between histone modification and prevalent chronic inflammatory skin diseases. We also review current and potential therapeutic approaches that revolve around modulating histone modifications. Finally, we investigated the prospects of research on histone modifications in the context of chronic inflammatory skin diseases, paving the way for innovative therapeutic interventions and improved patient outcomes.

## 1 Introduction

The epithelial tissues, comprising the outermost layer of an organism, serve as a protective barrier against environmental stressors, including physical, chemical, and microbial agents. Epithelial tissues are not only a physical barrier; they are also immunological organs that are activated in response to the attack of foreign agents and trigger different sets of transcriptional cascades that stimulate a specific type of immune response (1). The epithelial immune microenvironment is an important component in implementing this process; however, an aberration in this microenvironment may contribute to inflammatory skin diseases (2, 3).

Inflammatory skin diseases encompass a range of skin disorders characterized by the infiltration of inflammatory cells and a significant increase in inflammatory cytokines (4). The most prevalent inflammatory skin diseases include psoriasis, atopic dermatitis (AD), lupus, systemic sclerosis (SSc), contact dermatitis, lichen planus (LP), and alopecia areata (AA) The underlying causes of these diseases remain unclear. Genetic susceptibility has been proposed to partially explain the immune imbalances observed in patients with inflammatory skin conditions. Research indicates that defects in the filaggrin gene may increase the risk of allergic sensitization, atopic eczema, and allergic rhinitis (5). Another study discovered that the TYK2 gene’s loss-of-function missense variant rs34536443 is associated with decreased susceptibility to various autoimmune diseases in individuals with psoriasis (6). However, investigations involving monozygotic twins have shown that genetic factors do not comprehensively explain the underlying causes of inflammatory skin conditions (7, 8).

Epigenetic modifications, characterized by reversible alterations in gene expression, independent of DNA sequence changes, are manifested during various stages of development or in response to environmental stimuli. These modifications, commonly known as the “second code,” have gained increasing attention due to their significance in inflammatory skin diseases, specifically immune activation, T-cell polarization, and impairment of skin barrier function (9–13). In eukaryotic cells, the chromosomal DNA is organized into a compact structure known as chromatin (14). The chromatin comprises nucleosome subunits, consisting of a histone octamer made up of two copies each of the core histones H2A, H2B, H3, and H4, wrapped by 147 base pairs of DNA (15, 16). The nucleosomes are then condensed further into a higher-order chromatin structure when the linker histone H1 binds to the linker DNA. This condensation enables the storage of a significant amount of DNA, approximately six feet long when stretched from end to end, within a single cell (17–19). Dynamic chromatin remodeling significantly regulates various DNA-dependent biological processes, including RNA transcription, DNA replication, DNA repair, and chromosome condensation and segregation (20).

The chromatin structure can be classified into two categories according to its association with gene transcription. In a heterochromatic state, robust DNA-protein interactions induce highly condensed chromatin, characterized by limited interaction between transcription factors and the genome, which results in gene repression. Conversely, in a euchromatic state, DNA-protein interactions are diminished, resulting in loosely packed chromatin with an accessible structure that facilitates transcription factor binding to DNA, stimulating gene activation (21, 22). The core histones within the histone octamer exhibit a predominantly globular structure, except for their N-terminal “tails” that extend from the nucleosome. These N-terminal “tails” are rich in basic lysine and arginine residues and are subject to various modifications. Subsequently, these modifications result in conformational changes within the chromatin, enabling it to adopt relaxed or condensed states that either stimulate or inhibit transcription, respectively (23, 24). Histone modification has been shown to interact with the immune microenvironment and promote the occurrence and development of inflammatory skin diseases. A study reported that the α-KG–H3K9me3–BBOX1 axis is critical in the metabolic reprogramming of cluster of differentiation (CD)147, which is pivotal in glycolysis reprogramming during the pathogenesis of psoriasis (25). One of the histone deacetylases, SIRT1, potentially inhibits the apoptosis of keratinocytes induced by UVB in cutaneous lupus erythematosus (26). Another histone deacetylase, HDAC1, exhibits dysregulation in patients with AA and acne vulgaris (27). Some aberrant histone acetylation and methylation modifications were also found in PBMCs of patients with pemphigus vulgaris. These modifications may contribute to the pathological immune responses in the disease (28).

In this review, we described different types of histone modifications, analyzed their relationship with common chronic inflammatory skin diseases, and reviewed the therapeutic approaches currently employed for treating these conditions in association with histone modification. Additionally, we evaluated the prospects of utilizing histone modification in treating inflammatory skin diseases.

## 2 Different types of histone modifications and its function

### 2.1 Histone acetylation

**Figure 1:**
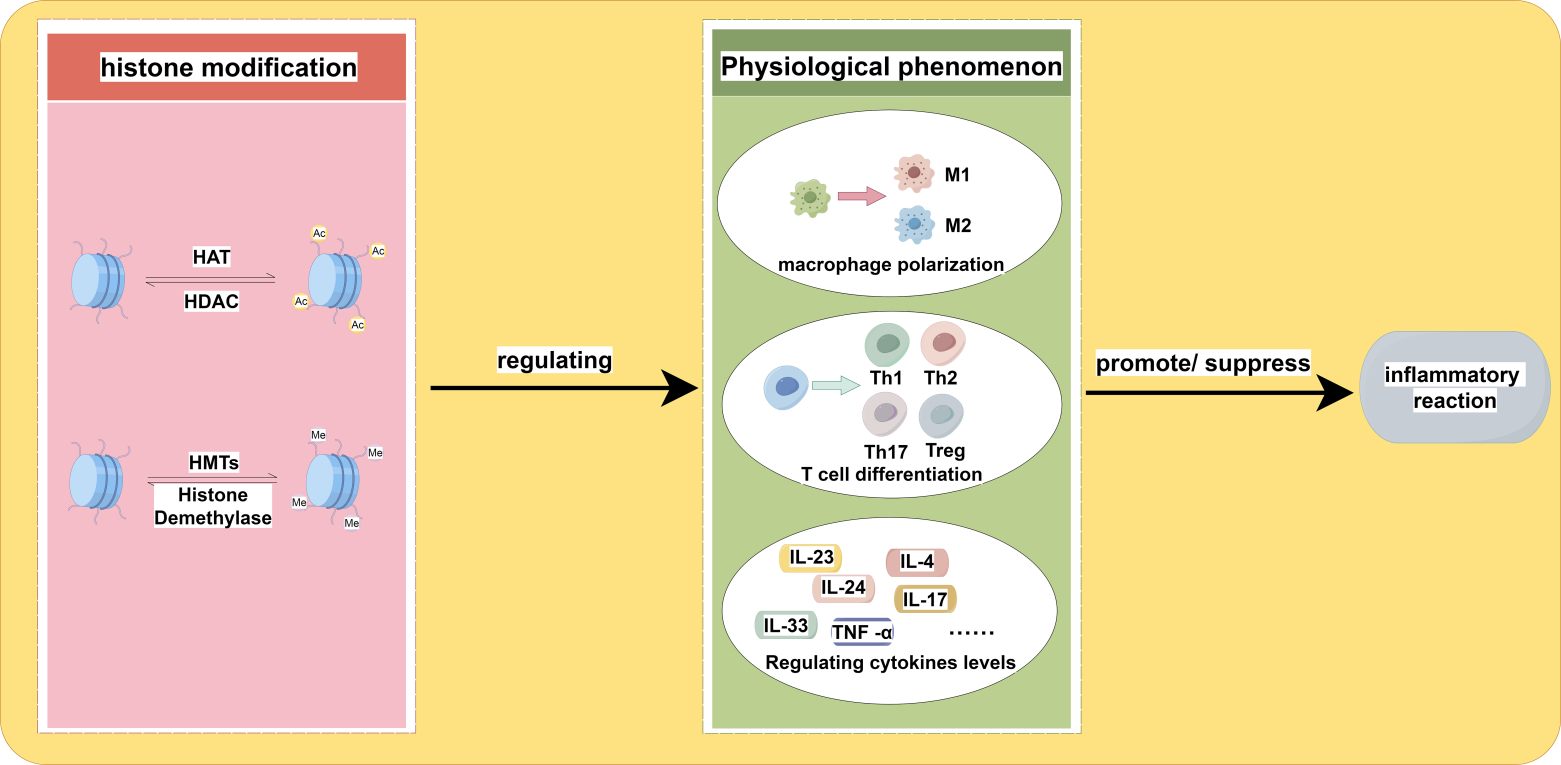
The impact of histone acetylation and histone methylation on the inflammatory response is examined. The up-regulation and down-regulation of these two types of histone modification play a role in macrophage polarization, T cell differentiation, and variation in cytokine levels. The specific locations and types of these modifications may lead to different directions of variation, ultimately influencing the regulation of the inflammatory response. The adjustment of histone modifications is mediated by relevant enzymes.

Histones typically contain amino acids with basic side chains that carry a positive charge, while genomic DNA carries a negative charge, creating an attractive force between the two (29). The process of histone acetylation primarily occurs at lysine residues, which neutralizes the positive charge of histones and reduces the interaction between nucleosomes and DNA. Consequently, histone acetylation results in a heterochromatic state, whereas histone deacetylation tends to result in a euchromatic state, significantly affecting the initiation and elongation of gene transcription (30, 31). The acetylation and deacetylation of histones are primarily regulated by histone acetyltransferases (HAT) and histone deacetylases (HDACs). HATs are protein complexes that facilitate the transfer of acetyl groups, including acetyl-CoA, onto the amino tails of histones (32). HDACs are categorized into four distinct classes: Class I (HDACs 1-3 and 8), Class II (HDACs 4-7, 9, and 10), and Class IV (HDAC11). HDACs require Zn^2+^ for their enzymatic activity, while Class III (Sirt1-7) HDACs are NAD^+^-dependent. These HDAC classes remove acetyl marks from histones and other protein substrates (17, 33). **Table 1** shows the classification and family of the enzymes involved in histone acetylation. Histone acetylation plays a significant role in the pathogenesis and progression of inflammatory diseases. Among the HAT proteins, KAT2A has been identified as one of them. The expression of KAT2A has been positively correlated with the immunopathology of inflammatory joint diseases in both patients with rheumatoid arthritis and mice with experimental arthritis. This correlation is attributed to the promotion of Il1b and Nlrp3 transcription through histone H3K9ac modification and the inhibition of NRF2 transcription repressor activity by KAT2A. Additionally, another type of HAT, CBP/p300, enhances cytokine expression by increasing the levels of H3K27a (34). Another type of HAT, known as CBP/p300, facilitates the expression of cytokines by upregulating the abundance of H3K27ac (35). Given the role of histone acetylation in modulating inflammatory responses, it is plausible to consider manipulating histone acetylation levels as a potential therapeutic approach for managing inflammatory disorders. **Figure 1** shows how histone acetylation influence inflammatory reaction.

**Table 1:** Enzymes involved in histone acetylation and histone methylation.

Classification	Family	Example
HAT	GNAT	GCN5, ELP3, HAT1, Hpa2
	p300/CBP	p300/CBP
	MYST	KAT5, KAT6B, KAT6A, KAT7, KAT8
	TAFII	TAFII250
HDACs	Class I	HDACs 1-3 and 8
	Class II	HDACs 4-7, 9 and 10
	Class III	Sirt1-7
	Class IV	HDAC11
HMTs	PRMTs	PRMT1-9
	HKMTs	KMT1-6
Histone Demethylase	LSD	KDM1A, KDM1B
	JMJD	JMJD1C, JMJD2D, JMJD3

### 2.2 Histone methylation

Histone methylation occurs at specific lysine or arginine residues located on histone’s N-terminal “tails” of histone (36). Compared to acetylation, methylation is a more intricate and nuanced process as it does not alter the charge in histone methylation, and both lysine and arginine residues can undergo multiple methylation states (37). Lysine residues, specifically H2BK5, H3K4, H3K9, H3K27, H3K36, H3K79, and H4K20, can undergo monomethylation, dimethylation, or trimethylation on the ϵ-amino groups of lysine residues. On the other hand, arginine residues, specifically H3R2, H3R8, H3R17, H3R26, and H4R3, can undergo monomethylation, symmetrical dimethylation, or asymmetrical dimethylation on their guanidinyl groups (23). Both the positioning of modified residues and the quantity of methyl groups are crucial for the functional consequences of histone methylation (38). The trimethylation of H3K4 (H3K4me3) at gene promoters and H3K36me3 across gene bodies results in open regions within chromatin and transcription activation. Conversely, H3K27me3 and H3K9me3 are commonly associated with gene silencing (17). In some conditions, monomethylation, including H3K9me1 and H3K27me1, is involved in the activation of transcription, while trimethylation at the same sites is associated with repression (17). **Table 1** displays the classification and family of the enzymes involved in histone methylation. The polarization of macrophages can be influenced by various types and levels of histone methylation, which in turn can impact the occurrence and progression of inflammation. The function of M1 macrophages is to produce pro-inflammatory cytokines, including TNF-α, IL-1β, IL-6, IL-12, and IL-23, through the expression of transcription factors, mainly the NF-κB. Macrophages adopt the M1 phenotype in response to pro-inflammatory cytokines secreted in response to persistent and severe inflammation or infection. However, if the M1 phase continues, it causes chronic inflammation, tissue damage, and loss of organ functions. Two kinds of histone methylation, H3K9me3 and H3K36me2, can negatively regulate the M1 phenotype (39, 40). On the contrary, a decrease of H3K27me3 may promote the M1 macrophage phenotype (41). Histone methylation is also very important in T-cell responses. The differentiation from naive CD4+ T cells to Th17 cells causes upregulation of H3K4me3 in the RORC promoter while decreasing H3K27me3. In contrast, the differentiation of Th1 cells is anticipated to contribute to the upregulation of H3K27me3 at the regulatory regions of RORC (42). These findings provide evidence suggesting that histone methylation plays a pivotal role in the inflammatory response. **Figure 1** shows how histone methylation influence inflammatory reaction.

### 2.3 Other modifications of histone

**Figure 2:**
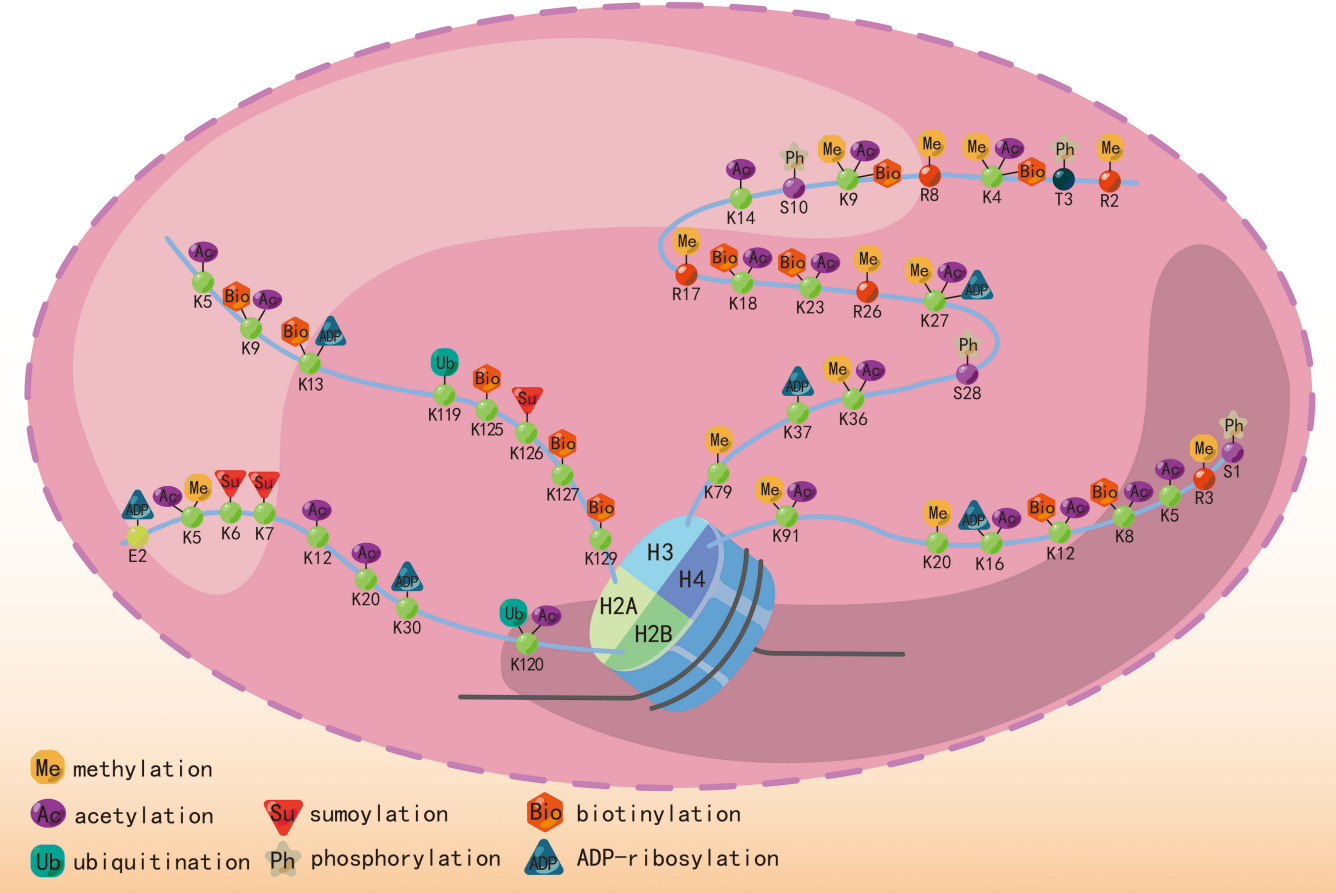
Different histone modifications, including methylation, acetylation, ubiquitination, phosphorylation, and sumoylation, and their position in the N-terminal “tails”.

The most studied histone modifications include acetylation and methylation, while other significant modifications include phosphorylation, ubiquitination, sumoylation, biotinylation, and ADP-ribosylation. Histone phosphorylation at serine and threonine residues contributes to chromatin relaxation (43) in various cellular processes, including transcription, mitosis, DNA repair, and apoptosis. Additionally, it enhances the effectiveness of histone acetylation (44, 45). Ubiquitin and small ubiquitin-like modifiers (SUMO) are conserved small proteins that undergo covalent attachment to an ϵ-amino group of a lysine residue on the histone (46, 47). Ubiquitination and sumoylation are associated with DNA double-strand break repair (48, 49). Histone biotinylation also occurs at lysine residues and may be involved in transcriptional repression (19, 50). ADP-ribosylation, catalyzed by ADP-ribose transferases using NAD^+^ as a cofactor, adds an ADP-ribose unit to an N-terminal chain. This process occurs in lysine, arginine, glutamic acid, and aspartic acid residues and is associated with DNA damage (51). **Figure 2** indicates different kinds of histone modifications and their position in N-terminal “tails”. The enrichment of H3 serine 28 phosphorylation was observed at induced genes in mouse macrophages stimulated with bacterial lipopolysaccharide, serving as a mediator for MSKs in the regulation of inflammatory response (52). USP38, a histone deubiquitinase, exhibits specificity in removing monoubiquitin from H2B at lysine 120. This process plays a role in coordinating inflammatory responses with the histone H3K4 modifier KDM5B (53). The process of histone ADP-riboyslation, mediated by PARP1, enhances the expression of inflammatory cytokines in microglia by promoting the accessibility of promoter DNA (54). The investigation of the association between inflammation and other kinds of histone modifications remains infrequently explored in scholarly literature.

## 3 Histone modifications in inflammatory skin diseases

### 3.1 Psoriasis

Psoriasis, a prevalent chronic inflammatory skin disease, exhibits a significant genetic predisposition and autoimmune pathogenic characteristics (55, 56). The presentation of psoriasis in patients varies in terms of morphology, distribution, severity, and course, although scaling papules and plaques are frequently observed. This condition is categorized as a “chronic, non-communicable, painful, disfiguring, and disabling disease for which there is no cure,” affecting a substantial population of over 60 million adults and children (57, 58). Psoriasis is characterized as an inflammatory skin disease mediated by Th17 cells. In the study conducted by Ovejero-Benito et al. (59), individuals with psoriasis had elevated levels of H3K4 compared to healthy individuals. Additionally, significant alterations in H3K27 were observed between individuals who responded positively to biological drugs and those who did not, specifically at the 3-month follow-up. H3K27 has been identified as a regulator of Th17 cell differentiation, while H3K4 has been associated with Th17 plasticity (60, 61). Additionally, a decrease in H3K9me2 in keratinocytes may contribute to the upregulation of IL-23, thereby playing a role in developing chronic inflammatory diseases dependent on the IL-23/IL-17 axis (62). A study of Rasheed et al. showed that the expression of Class III HDACs, specifically SIRT1, was reduced while SIRT6 was increased in psoriatic skin (63). Another study also observed an increase in HDAC1 and a decrease in SIRT1 levels in skin tissues (64). The transcription cofactor p300 is a histone acetyltransferase with histone acetylase activity. Liao et al. (65) reported that the transcription factor Wilms Tumor 1 regulates the expression of IL-1β by facilitating the binding of P300 to the IL-1β promoter, thereby contributing to the development of psoriasis. These publications demonstrated that histone acetylation and methylation play an important role in the occurrence and development of psoriasis. **Table 2** shows studies published on histone modification variation in psoriasis.

**Table 2:** Studies on histone modification in inflammatory skin disease in recent years.

Disease	Modification	Site	Study model	Results	Ref.
Psoriasis	Histone methylation	H3K9me3	Mice	CD147 is critical in metabolic reprogramming through the α-KG-H3K9me3- axis in the pathogenesis of psoriasis, indicating that epidermal CD147 is a promising target for psoriasis treatment.	(66)
	Histone methylation	H3K4me3, H3K4me1	Mice	Depletion of CD147 increased transcriptional expression and activity of γ-butyrobetaine hydroxylase (γ-BBD/), a crucial molecule for carnitine metabolism, by inhibiting histone trimethylations of H3K9.	(67)
	Histone acetylation	H3K27Ac	Human	In the most over-expressed genes in psoriasis, there is an enrichment of H3K27Ac. However, the loss of H3K27 acetylation modification does not correlate with decreased gene expression. GRHL appears to play an important role in the pathogenesis of psoriasis and, therefore, might be a new target for psoriasis therapeutics.	(68)
	Histone methylation	H3K27me3	Cell culture	EZH2 and H3K27me3 were over-expressed in the epidermis of psoriatic lesional skin compared to normal skin.	(69)
	Histone acetylation	H3K9Ac, H3K27Ac	Human&mice	Glutaminase 1-mediatedglutaminolysis was aberrantly activated in patients with psoriasis and in psoriasis-like mouse models, which promoted Th17 and γδ T17 (IL-17A-producing γδ T) cell differentiation through enhancement of histone H3 acetylation of the Il17a promoter, thereby contributing to the immune imbalance and development of psoriasis.	(70)
AD	Histone acetylation	/	Cell culture	TGase II, through interaction with NF-kB, induces expression of HDAC3 and snail, which in turn exerts transcriptional repression on E-cadherin to mediate allergic inflammation.	(71)
SSc	Histone acetylation/Histone methylation	H3K4me1, H3K27ac	Human	The SSc-associated haplotypes were enriched for H3K4me1/H3K27ac marks in monocytes.	(72)
	Histone methylation	H3K27me3	Human	SSc myofibroblasts *in vitro* and SSc skin biopsies *in vivo* display high levels of HOTAIR, a scaffold-long non-coding RNA known to direct the histone methyltransferase EZH2 to induce H3K27me3 in specific target genes.	(73)
	Histone acetylation	/	Human	CYR-61, epigenetically regulated by HDAC5, is a potent antifibrotic and proangiogenic mediator in SSc.	(74)
	Histone acetylation/Histone methylation	H3K4me3, H3K27	Human	1046 and 534 genomic loci showed aberrant H3K4me3 and H3K27ac marks, respectively, in SSc monocytes	(75)
SLE	Histone acetylation	/	Human	Upregulated SIRT1 inhibits the NLRP3 inflammasome to slow the progression of lupus nephritis by regulating NF-κB and ROS/TRPM2/Ca channels	(76)
	Histone methylation	/	Cell culture	JMJD3 could regulate B-cell differentiation by promoting naïve B-cell differentiation into CD27 B cells, and Blimp-1 and Bcl-6 also decreased after inhibitor treatment	(77)
	Histone acetylation/Histone methylation	H3K27Ac, H3K4me	Human	We observed many interactions in the TAD and strong enhancer markers (H3K4me1 and H3K27ac) near the two gene loci (online Supplementary Figure S8C). According to the Enhancer-Atlas2.0 database, the region was annotated as a super-enhancer in CD4+ T/CD8+ T cells	(78)
	Histone acetylation/Histone methylation	/	Mice	HDAC10 abundance was decreased in mouse macrophages in response to innate immune stimuli. It was reduced in peripheral blood mononuclear cells (PBMCs) from patients with systemic lupus erythematosus (SLE) compared with that in PBMCs from healthy donors.	(79)

### 3.2 Atopic dermatitis

AD is characterized by the presence of recurring eczematous lesions and severe itchiness and discomfort, resulting in sleep deprivation, reduced self-esteem, and impaired academic and occupational performance. Globally, AD affects approximately 7%−10% of adults and up to 25% of children with a significant genetic influence (80, 81). This allergic disease is primarily mediated by Th2 cells. Kwon’s study suggested that the interaction between mast cells, keratinocytes, and dermal fibroblast cells can potentially contribute to AD development. The presence of CXCL13 in the exosomes of mast cells in a pathological model was observed to enhance the expression of HDAC6 in both skin mast cells and dermal fibroblast cells.

Additionally, HDAC6 was found to negatively regulate MiR-9, thereby influencing the expression of SIRT1. The downregulation or inhibition of HDAC6 and SIRT1 demonstrated a suppressive effect on AD, as discussed in this article (82). Mesenchymal stem cells can interact with both the innate and adaptive immune systems and inhibit the activation of immune cells. These cells have been observed to be protective against AD, and this function has been linked to an increase in the expression levels of HDAC3 (83). Furthermore, the expression of miR-335, a potent inducer of keratinocyte differentiation, is abnormally diminished in AD-lesioned skin. This reduction is epigenetically regulated by histone deacetylases (84). The influence of histone modification to AD is mainly concentrated on acetylation. Publications on the histone methylation to AD are seldom to see. **Table 2** indicates studies published on histone modification variation in AD.

### 3.3 Systemic sclerosis

Scleroderma, or SSc, is an infrequent autoimmune disease affecting multiple systems, characterized by dysregulated innate and adaptive immunity resulting in extensive systemic fibrosis (85). Skin thickening has been extensively studied as a symptom of SSc because it facilitates diagnosis, and there has been a proven correlation between increased skin involvement and more severe internal organ damage, an unfavorable prognosis, and heightened disability (86). EZH2 is involved in T cell differentiation, EC-mediated angiogenesis, myofibroblast transformation, and tissue fibrosis in SSc (87–89). It has been reported that both EZH2 and H3K27me3 were elevated in SSc dermal fibroblasts and endothelial cells compared with healthy controls (90). JMJD3, a type of histone demethylase, is upregulated in SSc fibroblasts. This upregulation decreases H3K27me3 levels at the FRA2 promoter, which stimulates FRA2 expression and ultimately promotes SSc fibroblast activation (91). Ciechomska et al. reported that epigenetic alterations in monocytes can influence the pathogenesis of SSc by exerting either promotive or repressive effects on myofibrogenic differentiation (92). Histone acetylation also plays a significant role in SSc. In a study by Shin, ChIP-qPCR results showed an increase in H3K27 acetylation in SSc fibroblasts (93). The variation of histone modification is significant in fibroblasts and monocytes, which may be a valuable approach to understanding the development and changes of SSc. **Table 2** displays the studies published on histone modification variation in SSc.

### 3.4 Systemic lupus erythematosus

Systemic lupus erythematosus (SLE) is a chronic systemic autoimmune disease that may affect multiple tissues and organ systems, including cutaneous, renal, cardiopulmonary, musculoskeletal, neural, and hematologic systems (94). It is observed that nearly all patients with SLE experience cutaneous manifestations at some point during the progression of their disease (95). Currently, the treatment of SLE involves using glucocorticoids and immunosuppressive agents; however, the long-term prognosis for SLE remains unfavorable (96). Additionally, the regulation of SLE is significantly influenced by H3K27me3. Compared with healthy controls, lupus patients showed elevated levels of H3K27me3 in CD4+ T cells, a process facilitated by EZH2, a histone methyltransferase (97). Luo et al. observed a substantial increase in H3K27me3 at the HPK1 promoter in Tfh cells of SLE patients (98). In SLE mice, glomerular cells showed significantly upregulated HDAC6 and HDAC9 expression compared with healthy mice. Targeted inhibition of HDAC6 effectively reversed SLE-associated abnormalities by modulating the proportions of cells in the late pro- and early pre-B cell fractions and altering T cell differentiation, as indicated by increased Treg cells and decreased thymic DN T cells (99). Zhou et al. (100) showed a significant augmentation in H3 acetylation and H3K4me2 within the CD4+ T cells of individuals diagnosed with lupus. The anomalous alterations in histone modifications of CD4+ T cells potentially play a role in the pathogenesis of lupus by upregulating CD70 expression. More evidence suggested that histone modification take part in the abnormal differentiation of T cell in SLE individuals, which lead to the changes in pathological characteristics. The results of this study provided evidence that histone acetylation and methylation both play a role in regulating the immune microenvironment in SLE. **Table 2** lists the studies published on histone modification variation in SLE.

### 3.5 Contact dermatitis

Contact dermatitis, a prevalent inflammatory skin disease, is caused by direct exposure to environmental chemical substances that have the potential to irritate or cause allergenic reactions (101). These chemical substances facilitate the differentiation of T lymphocytes into Th2 cells, resulting in elevated levels of inflammatory cytokines and blood IgE (102). Additionally, the upregulation of IL-4 hinders the functionality of Th1 cells, resulting in reduced cell-mediated immunity and exacerbating the inflammatory response (103). Occupational contact dermatitis, a prominent subset of contact dermatitis, primarily affects individuals regularly exposed to water or irritating substances in their occupational settings (104). Hairdressers, construction workers, and healthcare professionals are particularly vulnerable to developing occupational contact dermatitis. Sonday et al. conducted a study involving 697 health workers in two tertiary hospitals in Southern Africa and reported that 12.3% of them exhibited a likelihood of contact dermatitis within the past 12 months (105). Additionally, contact with cosmetics and medical supplies was identified as a common source of contact dermatitis (106–108). A knockout experiment conducted in mice showed that HMGB1 exhibits anti-inflammatory properties in keratinocytes of contact dermatitis mice. The reduction in IL-24 expression is accomplished by inhibiting H3K4me3 binding to the promoter region of IL-24 (109). Additionally, another study indicated that the absence of Utx, a histone demethylase, could contribute to various T cell abnormalities and worsen the symptoms of contact dermatitis (110). Gaining insight into alterations in histone modification levels associated with contact dermatitis holds the potential to facilitate the development of novel preventive and therapeutic approaches aimed at enhancing the occupational and residential conditions of certain populations.

### 3.6 Lichen planus

LP is a chronic mucocutaneous disease characterized by the presence of reticular, purple papules and plaques on the skin, as well as white papules and erosions on mucous membranes, and progresses through a chronic relapsing course (111–113). LP primarily affects the skin and oral mucosa, affecting approximately 1%-2% of the general population (111). LP is more prevalent in middle-aged or older adults and women (114). LP is characterized by dysfunctional T cells that initiate epithelial cell apoptosis. Immune infiltration, primarily consisting of T cells, is a common occurrence in LP. Most cytotoxic clones observed in lichen planus lesions are CD8+ T cells, while most non-cytotoxic clones are CD4+ (115). In addition to contributing to the development of autoimmunity, these cytotoxic CD8+ T cells are essential for promoting apoptosis of oral mucosal basal cells (116). The World Health Organization (WHO) recognizes oral LP as a potentially malignant disorder (115). Some studies have found that histone acetylation take part in the process of LP. A study conducted on 66 patients with oral LP and 23 patients with cutaneous LP showed that H3K9 histone acetylation is more prevalent in both lesions, with no significant distinction between them (117). Another study reported that H3K9 histone acetylation acts as an epigenetic marker for the recurrence of oral LP (118). Jun et al. observed a decrease in histone H3 acetylation and an increase in HDAC activity in CD4+ T cells of oral LP patients, which may impact the synthesis of inflammatory cytokines. The level of histone H3 acetylation showed a negative association with IL-4 and MCP-1 production, while the expression of HDAC6 mRNA exhibited a positive correlation with MCP-1 production (119). Nevertheless, the relationship between LP and other kinds of histone modifications are required to be researched.

### 3.7 Alopecia areata

Alopecia areata (AA) is a prevalent autoimmune disorder that manifests as temporary and non-scarring hair loss. The affected regions can vary from small areas of baldness to complete hair loss on the scalp and face and, in some cases, extend to body hair (120). The global prevalence of AA is estimated at approximately 2%, with 0.27% prevalence in China (121). AA commonly affects a patient’s physical appearance and significantly impacts their health-related quality of life (121–123). Research indicates that individuals with AA may have a more than twofold increased risk of experiencing depression or anxiety disorders compared to the general population (124). AA is a disease that primarily affects the hair follicles; however, systemic immune activation and dysregulation of serum cytokine levels may be associated with this condition (125). AA patients typically exhibit perifollicular and intrafollicular infiltration, with CD4+ Th1 cells predominantly located near the hair follicle and CD8+ Tc1 cells within the follicular epithelium (126). Zhao et al. compared the epigenetic profiles of peripheral blood mononuclear cell (PBMC) samples from 25 AA patients and 20 healthy controls. AA patients exhibit a significant elevation in histone H3 acetylation levels and a significant reduction in histone H3 lysine-4 methylation levels compared with healthy controls. Additionally, there is a positive correlation between AA disease severity and the acetylation levels of histone H3. This study also observed an upregulation in the expression of p300, HDAC1, MLL, SETD7, EHMT2, KDM4C, and KDM5A, as well as a downregulation in the expression of HDAC2, HDAC7, KDM1A, KDM4A, and KDM4B. These results showed that epigenetic modification of PBMC may be involved in the pathogenesis of alopecia areata (127). In a study comprising a cohort of 25 individuals with patchy AA, 26 patients with acne vulgaris, and 25 healthy controls, the enzyme-linked immunosorbent assay (ELISA) data showed a decreased level of histone deacetylase 1 (HDAC1) in both AA and acne vulgaris patients (128). Additionally, the inhibition of SIRT1 enhanced the synthesis of Th1 cytokines (IFN-γ and TNF-α) IFN-inducible chemokines (CXCL9 and CXCL10). It facilitated T cell migration in hair follicles’ outer root sheath cells. Conversely, the activation of SIRT1 suppressed the autoreactive inflammatory reactions. The antagonistic impact of SIRT1 on the immune response was mediated via the deacetylation of NF-κB and the phosphorylation of STAT3. Thus, SIRT1 downregulation may contribute to AA development (129). The variation of histone acetylation is observed in AA patients, which provides new ideas for the treatment of AA.

## 4 Therapeutic method and potential therapeutic method related to histone modifications

### 4.1 Short-chain fatty acids and their derivative

The occurrence and progression of inflammatory skin diseases are frequently accompanied by gut microbiota alterations, which serve multiple functions (130–133). The gut microbiota produces a wide range of short-chain fatty acids (SCFAs) through the fermentation of non-digestible carbohydrates, including dietary fiber (134). SCFAs are fatty acids with carbon backbones ranging from one to six, and the predominant SCFAs found in the intestines, with concentrations exceeding 100 mM, are propionate, acetate, and butyrate (134, 135). SCFAs are produced by two predominant bacterial groups, with acetate and propionate primarily generated by the Bacteroidetes phylum, while butyrate is mainly produced by the Firmicutes phylum (136). An animal model study showed that mice with intestinal microbiota exhibited higher histone acetylation levels than germ-free mice. Conversely, when SCFAs were administered through drinking water, histone acetylation in germ-free mice increased significantly to a higher level compared with the microbiota group. This study proves that gut microbiota regulates histone modification through SCFA metabolism (137). Another study showed that SCFAs produced by Propionibacterium acnes can inhibit the activity of HDAC, thereby contributing to the development of inflammatory responses (27). Short-chain fatty acids (SCFAs) are produced through the metabolic processes of gut bacteria and exert an impact on the development of inflammatory skin conditions by inhibiting histone deacetylase (HDAC). This observation underscores the intricate nature of the etiology of inflammatory skin diseases and presents a novel therapeutic avenue for their management. In contrast, imiquimod reduces the suppressive activity of Treg, resulting in the upregulation of IL-17 and IL-6 and the downregulation of IL-10 and FOXP3. Consequently, this effect helps develop a psoriasis-like skin inflammation model. One specific SCFA, butyrate, can reverse these processes by inhibiting HDACs (138). SCFAs represent a promising therapeutic target for inflammatory skin diseases due to their involvement in the regulation of histone modification. Krautkramer et al. (137)reported that administering SCFAs through water to germ-free mice can replicate gut colonization and its impact on histone modification. The combined treatment of sodium butyrate, one of the SCFAs, and the EGFR inhibitor PD153035 has enhanced keratinocyte differentiation (139). Another study showed that administering either injected or topically applied sodium butyrate onto the ears of mice sensitized to 2,4,6-trinitro-1-chlorobenzene significantly reduced the contact hypersensitivity reaction (140). As histone deacetylase inhibitors, butyrate and propionate upregulate miRNAs that suppress AID expression, thereby modulating autoantibody responses in lupus-prone MRL/Faslpr/lpr mice (141). Luo et al. reported that butyrate can suppress IL-33 expression in keratinocytes infected with S. aureus by inhibiting HDAC3 (142). These findings suggest that the modulation of SCFAs on histone modification can potentially treat inflammatory skin diseases; however, additional research is required on autoimmune disorder therapies.

### 4.2 HDAC inhibitors

HDAC inhibitors modulate gene expression by inhibiting the removal of acetyl groups from the N-terminal tails of histones through binding to HDAC (13, 143). Extensive research has shown the potential of HDAC inhibitors in antineoplastic therapy (144–146). Additionally, the antiproliferative and anti-inflammatory properties of HDAC inhibitors have prompted an active investigation into their efficacy for treating inflammatory skin diseases (13). Trichostatin A (TSA) is one of this context’s most extensively studied HDAC inhibitors. A study by Kim showed that TSA treatment resulted in a decreased IL-4 production and an increased T reg cells population, effectively suppressing AD-like skin lesions in NC/Nga mice treated with DNFB under SPF conditions (147). Additionally, TSA can reverse the aberrant expression of multiple genes associated with the immunopathogenesis of SLE patients (148). Mohammadi’s (149) study showed that sodium valproate inhibits HDAC in SLE patients, resulting in immunomodulatory effects on macrophages. Additionally, a study on 10 AD patients and 6 healthy individuals showed that the HDAC inhibitor belinostat restored epidermal miR-335 expression and rescued the impaired skin barrier in AD (84). Souliotis et al. reported that administering vorinostat resulted in hyperacetylation of histone H4, chromatin decondensation, improved DNA repair efficiency, and decreased apoptosis among patients with SLE. Consequently, genes involved in DNA damage repair and signaling pathways were significantly under-expressed, while genes associated with apoptosis were significantly over-expressed (150). Additionally, panobinostat treatment in SLE mice significantly reduced autoreactive plasma-cell numbers, autoantibodies, and nephritis, while other immune parameters remained largely unaffected. The findings indicate that panobinostat has the potential to be a therapeutic option for autoimmune conditions driven by B-cells without causing significant long-term negative effects on B-cell memory (151). Clinical applications for systemic lupus erythematosus were demonstrated by suberoylanilide hydroxamic acid in two distinct mouse model experiments (152, 153). Different types of HDAC inhibitors have been shown to have an impact on inflammatory skin diseases, thereby offering novel support for the management of such conditions.

### 4.3 Bromodomain and extraterminal protein inhibitor

**Figure 3:**
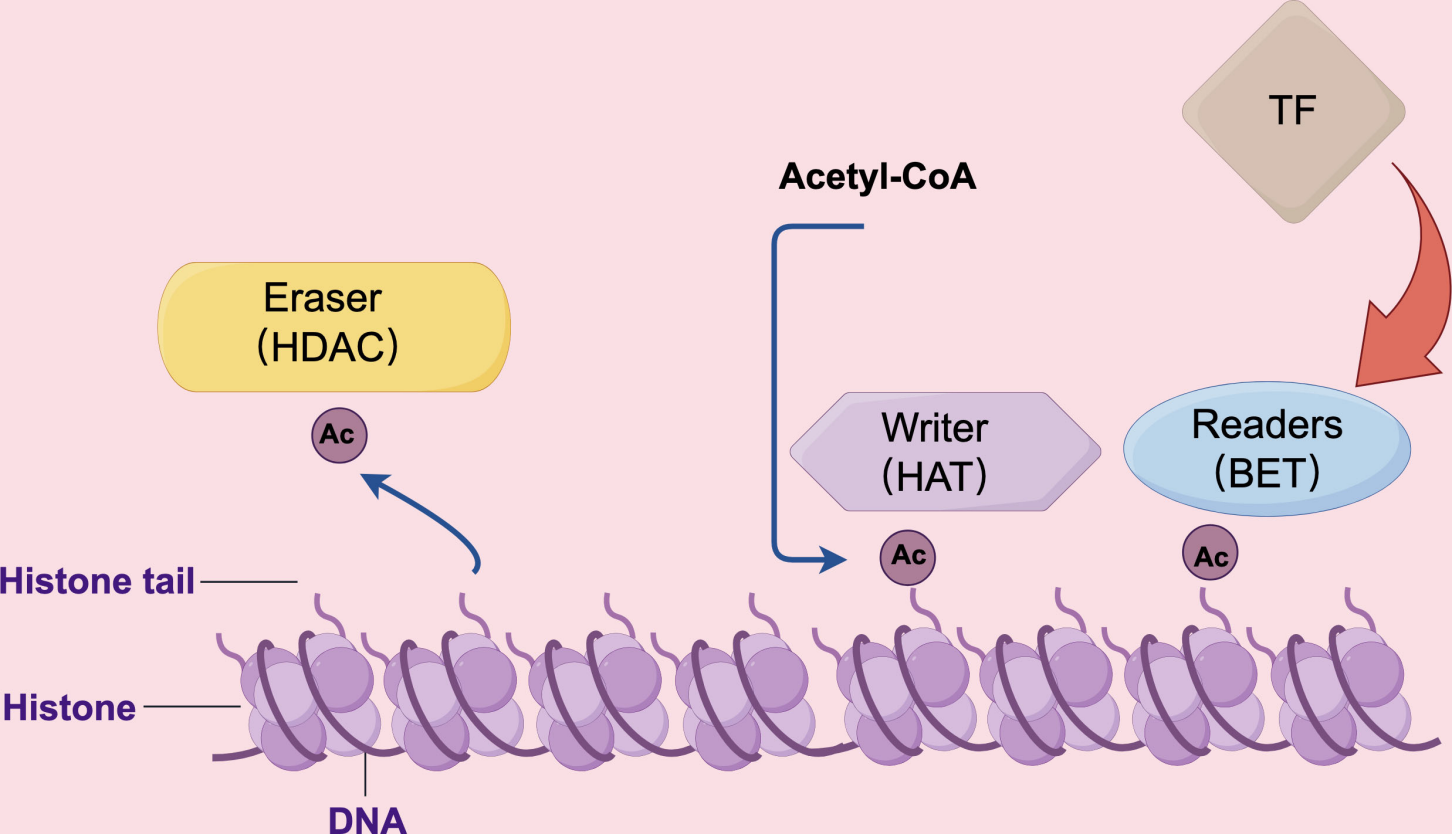
The HAT, HDAC, and BET proteins perform distinct roles in the regulation of gene transcription. HAT proteins function as “writers” by modifying lysine residues with acetyl groups using acetyl CoA. Conversely, BET proteins act as “readers” by binding to acetylated lysine residues and facilitating the recruitment of transcription factors. HDAC proteins, on the other hand, function as “erasers” by deacetylating lysine residues, thereby reducing the frequency of transcription events.

Bromodomain proteins, a group of evolutionarily conserved motifs involved in protein-protein interactions, have been found to recognize acetylated lysine residues on histones and play a significant role in chromatin remodeling (154). Bromodomain proteins are characterized by the presence of four left-handed bundle α-helices, which are arranged in a manner that creates two distinct interhelical αZ-αA and αB-αC loop regions. These loop regions contribute to the formation of a hydrophobic pocket. The primary function of this hydrophobic pocket is to facilitate the recognition of acetylation modifications on lysine residues (155). The bromodomain and extra-terminal domain (BET) family of proteins is most widely studied among bromodomains in cellular biology. BET proteins consist of two tightly packed BRDs, BD1 and BD2, and an extra-terminal domain (ET) located at the carboxy terminus. The ET region is also highly conserved, consisting of 80 amino acids (156). This domain’s primary role is to interact with various cellular proteins, including histone-lysine N-methyltransferase and Jumonji domain-containing 6 (157, 158). As is shown in **Figure 3**, known as “chromatin readers,” BET proteins play a crucial role in numerous biological processes, including chromosomal architecture, DNA replication, DNA damage repair, and transcriptional regulation (156). JQ1, one of the BET inhibitors, has exhibited significant efficacy in suppressing multiple inflammatory and autoimmune diseases. JQ1 mitigates lupus in MRL-lpr mice by suppressing BAFF, pro-inflammatory cytokines, and autoimmunity. This finding suggests the therapeutic potential of JQ1 in treating lupus disease (159). Another study involving mice and cell culture showed that BRD4 inhibitors exhibited superior antifibrotic effects compared to other BET inhibitions (160). Additionally, it has been reported that OTX015 and ABBV075 can reduce the severity of imiquimod-induced psoriasis in mice. ABBV075 achieves the same pharmacodynamics at a dosage one-tenth of that required for OTX015 (161). Sato et al. (162) designed and synthesized various pyrido-benzodiazepinone derivatives, among which one exhibited the most effective therapeutic effect in treating imiquimod-induced psoriasis mice. Currently, the primary emphasis of clinical investigation concerning BET inhibitors lies in their application for anti-tumor therapy. Nevertheless, considering their involvement in transcription subsequent to histone modification, it is anticipated that these pharmaceutical agents will find utility in the management of inflammatory skin disorders.

## 5 Discussion and expectations

The epithelial immune microenvironment of the skin is a dynamic network composed of keratinocytes, immune cell subpopulations, cytokines, and metabolites. The disorder of the epithelial immune microenvironment may contribute to inflammation reactions. Inflammation has long been recognized as a defensive mechanism triggered by infections or injuries characterized by rubor, calor, swelling, and pain. It is an immune cell-mediated biological response that occurs when the body is stimulated by microorganisms, pathogens, damaged cells, and various internal and external environmental factors (163). Inflammation serves as a crucial defense mechanism in preserving the well-being of the host. It stimulates the activation of the immune system to eliminate the pathogen, initiates the healing process, reduces the severity of tissue damage and infection caused by the stimulus, and ultimately counteracts the threat while restoring homeostasis (164). Conversely, inflammation frequently coincides with heightened acidity in the affected area. Prolonged persistence of unresolved inflammation may result in the development of chronic inflammation and tissue deterioration, which can further increase the prevalence of inflammatory disorders and skin diseases.

Inflammation is an intricate and sequential phenomenon requiring synchronized involvement of various cellular and tissue components. The intercellular communication within inflammatory systems is primarily orchestrated by cytokines, encompassing protein, peptide, or glycoprotein messenger molecules (165). While inflammation shares similarities with the nervous system regarding sensory and effector pathways, inflammatory cells can migrate to any location within any tissue (166). Consequently, there may be inconsistencies in the etiology and distribution of inflammatory diseases, occasionally resulting in systemic abnormalities. The inflammatory cascade comprises cytokines, chemokines, growth factors, and other peptides synthesized by skin keratinocytes and immune cells. The expression of these components is controlled by epigenetic modifications (13). The influence of histone modification on inflammatory reaction often focuses on two sides: immunocyte and epithelial cells. Section two demonstrates that the modulation of histone modification can impact the polarization of macrophages. Additionally, histone modification exerts an influence on the differentiation and function of T cells. These mechanisms have the potential to either facilitate or impede inflammatory responses, thereby influencing the development of inflammatory skin conditions. It is worth noting that both histone methylation and histone acetylation play a role in this regard. For instance, the administration of VPA has been shown to inhibit the activity of HDAC, modulate macrophage polarization, increase the expression of anti-inflammatory cytokines, and enhance the immune response against inflammation in patients with SLE (149). In a separate study, the inhibition of SIRT1 was found to stimulate the secretion of Th1 cytokines, IFN-inducible chemokines, and T cell migration in ORS cells, thereby contributing to the development of AA (129). The significance of epithelial cells in the regulation of immunity has gained increasing recognition (167). Keratinocytes, being the principal epithelial cell in the skin, exert influence on immune responses through two mechanisms: direct expression of cytokines and antimicrobial peptides, as well as indirect functions as a barrier separating the environment from classical immunocytes (12). The reduction of H3K9me2 in keratinocytes within psoriatic lesions demonstrates a positive association with IL-23 expression, potentially contributing to the development of psoriasis (65). The inhibition of HDAC3 by butyrate leads to a decrease in IL-33 expression and subsequently alleviates skin inflammation in a mouse model resembling atopic dermatitis (142). The aforementioned findings suggest that the modulation of keratinocytes through epigenetic mechanisms has the potential to impact the development and progression of inflammatory skin diseases.

However, the complexity of this regulatory mechanism arises from the fact that similar epigenetic modifications can elicit diverse responses in various cell types (168, 169). Histone acetylation induces a state of open chromatin that facilitates the binding of transcription proteins, potentially leading to elevated expression of pro-inflammatory cytokines or suppression of inflammatory mediators in various cell types. Typically, HDAC inhibitors are considered crucial in anti-inflammatory approaches; however, Sanford et al. reported contradictory findings (170). SCFAs, acting as HDAC inhibitors, possess anti-inflammatory properties in myeloid-derived cells; they may disrupt the epidermis’ tolerance to inflammatory stimuli. Applying SCFAs on the skin surface and their subcutaneous administration may yield contrasting outcomes. Another study on cell cultures revealed that SCFAs can exhibit pro-inflammatory effects under specific circumstances (27). The elimination of Sirt2 was observed to exacerbate psoriasiform skin inflammation, whereas the reintroduction of Sirt2 through genetic means reduced disease severity (171).

The consideration of drug side effects, particularly those related to histone modification, is crucial in the treatment of inflammatory skin diseases. It has been reported that hematologic toxicity represents a significant concern in certain HDAC inhibitor drugs (172). Prolonged treatments with HDAC inhibitors have been associated with various adverse events, including leucopenia, neutropenia, thrombocytopenia, anemia, peripheral sensory neuropathy, fatigue, vomiting, and myalgia (173, 174). Therefore, a comprehensive evaluation of the side effects associated with HDAC inhibitor therapy is required (175–177). Developing new HDAC inhibitors aims to achieve good efficacy while minimizing side effects. HDAC inhibitors can be categorized into four groups: hydroxamates, benzamide derivatives, cyclic peptides, and aliphatic acids. Benzamide derivatives are believed to possess lower toxicity than hydroxamates due to their isotype-selective nature as opposed to being pan-HDAC inhibitors (178). Bollmann et al. have successfully developed a novel and highly selective HDAC inhibitor called YAK540. The combined administration of YAK540 and the proteasome inhibitor bortezomib shows potential as a promising strategy against leukemias, as it enhances the anticancer efficacy while minimizing the overall toxicity associated with HDAC inhibitors (179). A novel drug delivery system utilizing oxygen-containing nanosomes has been developed to efficiently transport HDAC inhibitors to dormant HIV-infected cells. Incorporating oxygen nanosomes in this study alleviates drug toxicity and regulates the rate of drug release (180). The administration of BET inhibitors also presents side effects. The most commonly observed treatment-related adverse events include thrombocytopenia, anemia, fatigue, and gastrointestinal complications (181). These side effects arise due to the ability of BET inhibitors to target any proteins containing bromodomains (182). Therefore, a BET inhibitor with enhanced selectivity and reduced side effects has been developed compared to pan BET inhibitors. GSK778 and dBET57 exhibit BD1-selectivity, while GSK046, ABBV-744, and SJ432 exhibit BD2-selectivity. Conversely, AZD5153 is a bivalent BET inhibitor specifically targeting both BD1 and BD2 of Brd4 (183–187). Although most of these studies do not pertain to inflammatory skin disease, it is anticipated that novel inflammatory skin disease epigenetic therapies with low toxicity and high efficacy will be developed soon.

Our assay aims at summarize the relationship between inflammatory skin disease and one type of epigenetics: histone modification. For it is a huge family of epigenetic modification and various function can be achieve by this kind of phenomena. Therapeutic method and potential therapeutic method related to histone modification was introduced by this assay. Many recently published literature has also been reviewed in this assay. In conclusion, histone modification is a crucial biological process in various inflammatory skin diseases. The studies on variations in histone modification during the pathogenesis of these diseases continue accumulating, and our understanding of their onset and progression is progressively expanding. Developing novel therapeutic approaches targeting histone modification, such as next-generation drugs and combination therapy methods, has become a pivotal area of research interest.

## Author contributions

LZ: Conceptualization, Writing – original draft. RC: Visualization, Writing – original draft. ZT: Conceptualization, Writing – original draft. FM: Visualization, Writing – review & editing. XS: Writing – review & editing. QZ: Funding acquisition, Writing – review & editing. ZC: Funding acquisition, Writing – review & editing.
